# A Statistical Method to Distinguish Functional Brain Networks

**DOI:** 10.3389/fnins.2017.00066

**Published:** 2017-02-14

**Authors:** André Fujita, Maciel C. Vidal, Daniel Y. Takahashi

**Affiliations:** ^1^Department of Computer Science, Institute of Mathematics and Statistics, University of São PauloSão Paulo, Brazil; ^2^Department of Psychology and Princeton Neuroscience Institute, Princeton UniversityPrinceton, NJ, USA

**Keywords:** random graph, analysis of variance, graph spectrum, network science, functional connectivity, anogva

## Abstract

One major problem in neuroscience is the comparison of functional brain networks of different populations, e.g., distinguishing the networks of controls and patients. Traditional algorithms are based on search for isomorphism between networks, assuming that they are deterministic. However, biological networks present randomness that cannot be well modeled by those algorithms. For instance, functional brain networks of distinct subjects of the same population can be different due to individual characteristics. Moreover, networks of subjects from different populations can be generated through the same stochastic process. Thus, a better hypothesis is that networks are generated by random processes. In this case, subjects from the same group are samples from the same random process, whereas subjects from different groups are generated by distinct processes. Using this idea, we developed a statistical test called ANOGVA to test whether two or more populations of graphs are generated by the same random graph model. Our simulations' results demonstrate that we can precisely control the rate of false positives and that the test is powerful to discriminate random graphs generated by different models and parameters. The method also showed to be robust for unbalanced data. As an example, we applied ANOGVA to an fMRI dataset composed of controls and patients diagnosed with autism or Asperger. ANOGVA identified the cerebellar functional sub-network as statistically different between controls and autism (*p* < 0.001).

## 1. Introduction

Graphs are widely used to represent interactions such as functional connectivity among brain regions (Bullmore and Sporns, 2009), social networks (Scott, 2012), and molecular interactions (Barabási and Oltvai, 2004). Once interaction graphs are obtained, a common problem is to verify if graphs of different populations are comparable or not. Standard approaches are based on algorithms to determine isomorphism—one-to-one correspondence—or how close to isomorphism are different graphs. For example, if the vertices of the graphs are labeled, one may count how many times a certain edge is present in each population. Otherwise, one may try to find an isomorphic sub-network, which problem is known to be NP-complete. Both strategies are not the most adequate given that real-world interaction graphs are heterogeneous and present intrinsic randomness. For example, functional brain networks of different individuals are structurally different, even belonging to the same group. Even networks from the same subject can change if measured on different times. Notice that in both examples, algorithms based on isomorphism will falsely discriminate graphs belonging to the same group or state. One solution for this problem is to assume that real-world graphs are generated by probabilistic processes (random graph models) and then test whether populations of graphs are generated by the same random graph model. However, the model that generated the graph is rarely known in practice. Thus, the first step to discriminate random graphs is to identify highly distinctive features across different graph models.

The spectrum (set of eigenvalues) of the adjacency matrix describes several structural properties of a random graph, such as the number of walks, diameter, and cliques (Van Mieghem, 2010). Therefore, it is a natural candidate to distinguish graphs generated by different processes (Van Mieghem, 2010). Indeed, in general, the graph spectrum is a better and more general characterization of complex networks in comparison to other features, such as the number of edges, degree, and centrality measures (Takahashi et al., 2012). By analyzing the graph spectrum (Takahashi et al., 2012) defined the concept of graph spectral entropy and developed statistical methods on graphs for (i) model selection, (ii) parameter estimation, and (iii) a hypothesis test to discriminate whether two populations of graphs are generated by the same random graph model and parameters. These methods are important from a methodological viewpoint because it provided formal methods for the statistical inference using graph samples. From a practical perspective, these methods were essential to identify novel brain sub-networks associated with attention deficit hyperactivity disorder (ADHD) (Sato et al., 2013) and autism spectrum disorder (ASD) (Sato et al., 2015). However, the random graph comparison method introduced in Takahashi et al. (2012) cannot verify simultaneously whether three or more groups of graphs are generated by the same random graph model. For example, it is not possible to simultaneously test the equality of the functional brain networks of controls, autism, and Asperger subjects. One possible solution would be to compare the groups in a pairwise manner, nevertheless these methods in general give an inadequate control of type I error. Here, we introduce a statistical method to discriminate two or more populations of graphs simultaneously, namely ANOGVA (Analysis of Graph Structure Variability). Intuitively, if the original test proposed by Takahashi et al. (2012) is equivalent to a *t*-test, our proposed test is equivalent to the analysis of variance (ANOVA) (Fisher, 1918).

We illustrate the performance of ANOGVA through simulation studies and demonstrate the power of the test for identifying small differences in the parameters of the random graph models. We also applied our method to study the whole brain functional magnetic resonance imaging (fMRI) data of 908 controls and patients diagnosed with autism or Asperger.

## 2. Materials and methods

Let us first formalize our problem. Given *k* populations of graphs *g*_1_, *g*_2_, …, *g*_*k*_ where each population *g*_*i*_(*i* = 1, …, *k*) is composed of |*g*_*i*_| graphs, we would like to verify whether the graphs of the *k* populations were generated by the same probabilistic process, i.e., by the same random graph model [e.g., Erdös-Rényi (Erdös and Rényi, 1960), Watts-Strogatz (Watts and Strogatz, 1998), and Barabási-Albert (Barabasi and Albert, 1999) random graph models] and set of parameters. First we will describe the graph spectrum. Based on the graph spectrum, we will define the Kullback-Leibler divergence between two random graphs that will be used to define the ANOGVA statistics.

### 2.1. Graphs and graph spectrum

A graph is a pair of sets *G* = (*V, E*) where *V* is a set of *n* vertices and *E* is a set of *m* edges that connect two vertices of *V*. A random graph *g* is a family of graphs, where the members of the family are generated by some probability law.

An undirected graph *G* with *n* vertices can be represented by its *n* × *n* adjacency matrix **A** where **A**_*ij*_ = **A**_*ji*_ = 1 if vertices *i* and *j* are connected, and 0 otherwise. The spectrum of *G* is the set of eigenvalues (λ_1_ ≥ λ_2_ ≥ … ≥ λ_*n*_) of the adjacency matrix **A**. For an undirected graph, the adjacency matrix is symmetric, and thus, its eigenvalues are real (Strang, 2011).

Given a set of random graphs *g* generated by the same probability law, the set of eigenvalues Λ are random vectors. Let δ be the Dirac delta function and the brackets “<>” indicate the expectation with respect to the probability law of the random graph, the spectral distribution of a random graph *g* is defined as:

(1) $$\begin{array}{r} \rho_{g}\left(\lambda \right)=\underset{n\to \infty }{\lim}<\frac{1}{n}\overset{n}{\underset{j=1}{\sum }}\delta \left(\lambda -\lambda_{j}/\sqrt{n}\right)>. \end{array}$$

The spectral distribution is directly associated with the structural features of the graphs (Albert and Barabási, 2002) and can be considered as a fingerprint of the random graph, where each random graph model is associated with a specific spectral distribution ρ_*g*_ (Van Mieghem, 2010).

### 2.2. Kullback-Leibler divergence

Once the spectral distribution of a graph is defined, we can describe a measure of similarity between two spectral distributions. If two spectral distributions are different, then the respective graphs should be different.

Let ρ_*g*_1__ and ρ_*g*_2__ be the spectral distributions of random graphs *g*_1_ and *g*_2_, respectively. If the support of ρ_*g*_2__ contains the support of ρ_*g*_1__, the Kullback-Leibler (KL) divergence between two spectral distributions ρ_*g*_1__ and ρ_*g*_2__ is (Kullback and Leibler, 1951):

(2) $$\begin{array}{r} KL\left(\rho_{g_{1}}|\rho_{g_{2}}\right)=\int_{-\infty }^{+\infty }\rho_{g_{1}}\left(\lambda \right)\log\frac{\rho_{g_{1}}}{\rho_{g_{2}}}d\lambda , \end{array}$$

otherwise, *KL*(ρ_*g*_1__|ρ_*g*_2__) = +∞ (we assume $0\;\log\frac{0}{0}=0$).

For Equation (2), ρ_*g*_2__ is called the reference measure. The KL divergence is non-negative and zero if and only if ρ_*g*_1__ and ρ_*g*_2__ are equal.

### 2.3. Estimation of the spectral density

To estimate the spectral density (${\hat{\rho }}_{g}$), we use the same procedure described by Takahashi et al. (2012). First we compute the eigenvalues of the adjacency matrix of the graph. Then, we apply a Gaussian kernel regression using the Nadaraya-Watson estimator (Nadaraya, 1964) for regularization of the estimator. Finally, we normalize the density to obtain the integral below the curve equal to one. In general, smaller sample and/or graph sizes require larger bandwidth and smaller bin numbers for the smoothing kernels. The opposite holds for larger sample and/or graph sizes. The exact bandwidth size and bin number that maximize the statistical power depends on the data and the alternative hypotheses, but some rules of thumb exist in the literature that have shown good performance in our simulations. The bandwidth of the kernel is chosen as $\frac{\lambda_{1}\;-\;\lambda_{n}}{\text{number of bins}}$ (Sain, 1996), where the number of bins is selected by using the Sturge's criterion (Sturges, 1926). Type I errors are controlled by our bootstrap procedure discussed below for any choice of bandwidth size and bin number.

### 2.4. Analysis of graph structure variability—ANOGVA

We are now able to describe ANOGVA. Given *k* populations of graphs *g*_1_, *g*_2_, …, *g*_*k*_, the test consists of verifying if all populations of graphs were generated by the same random graph model. For this, we test if all the spectral distributions are equal.

Let ${\hat{\rho }}_{g_{1}},{\hat{\rho }}_{g_{2}},\ldots ,{\hat{\rho }}_{g_{k}}$ be the estimated spectral distributions of populations of graphs *g*_1_, *g*_2_, …, *g*_*k*_, respectively, where ${\hat{\rho }}_{g_{i}}$ (*i* = 1, …, *k*) is the average of the graphs spectra in population *g*_*i*_. Also, set ${\hat{\rho }}_{g_{M}}=\frac{\overset{k}{\underset{i=1}{\sum }}{\hat{\rho }}_{g_{i}}}{k}$. The support of ${\hat{\rho }}_{g_{M}}$ includes the support of ${\hat{\rho }}_{g_{i}}$ for any *i*. Formally, we test:

H_0_: *KL*(ρ_*g*_1__, ρ_*g*_*M*__) = *KL*(ρ_*g*_2__, ρ_*g*_*M*__) = … = *KL*(ρ_*g*_*k*__, ρ_*g*_*M*__) = 0, i.e., the graphs from *g*_1_, *g*_2_, …, *g*_*k*_ are generated by the same random graph model (the spectral distributions are equal).

H_1_: “At least one population of graphs is generated in a different manner”.

We will use the statistic $\Delta =\overset{k}{\underset{i=1}{\sum }}KL\left({\hat{\rho }}_{g_{i}},{\hat{\rho }}_{g_{M}}\right)$ to build the test statistic. This statistic is the generalization of the Jensen-Shannon divergence (Jensen, 1906; Shannon, 1948) for *k* > 2. Under the null hypothesis, we expect small Δ, while large Δ suggests a rejection of the null hypothesis. The exact or asymptotic distribution of Δ under the null hypothesis is not known; therefore, we use a computational procedure based on the permutation test to construct the empirical distribution. The steps for the permutation test is as follows:

1. Construct permuted samples $g_{i}^{*}$, for *i* = 1, …, *k* by resampling (without replacement) |*g*_*i*_| graphs from the entire dataset {*g*_1_∪*g*_2_∪ … ∪*g*_*k*_}.
2. Calculate ${\hat{\rho }}_{g_{i}^{*}}$ for each $g_{i}^{*}$ (*i* = 1, …, *k*).
3. Calculate ${\hat{\Delta }}^{*}=\overset{k}{\underset{i=1}{\sum }}KL\left({\hat{\rho }}_{g_{i}^{*}},{\hat{\rho }}_{g_{M}}\right)$.
4. Repeat steps 1 to 4 until the desired number of replications is obtained.
5. The *p*-value for the observed statistic $\hat{\Delta }$ is the fraction of times ${\hat{\Delta }}^{*}$ obtained in the permuted dataset is at least as large as $\hat{\Delta }$ estimated in the original dataset.

**Figure 1:**
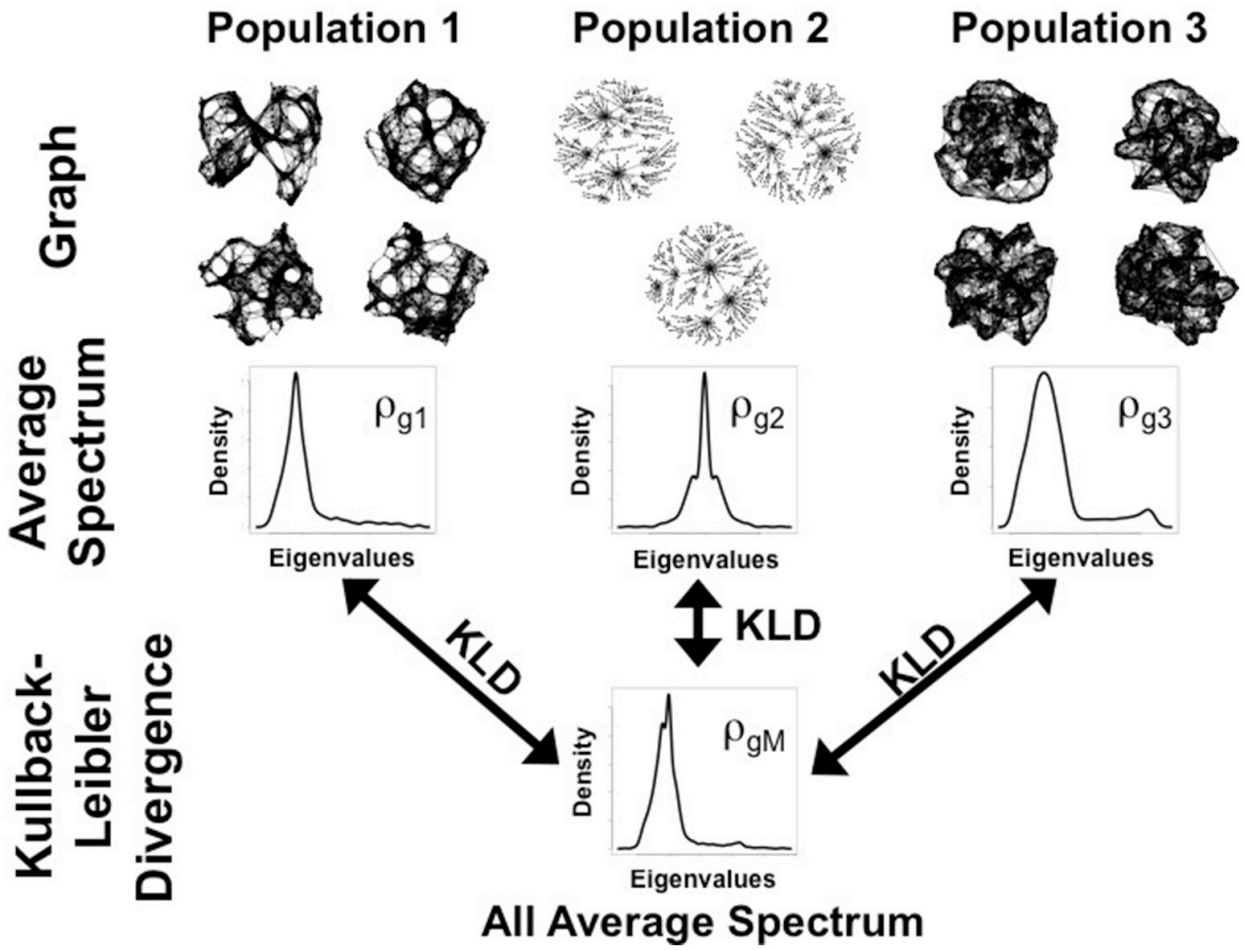
**Schema of ANOGVA analysis**. In this example, *k* = 3 populations of graphs are tested to verify whether they were generated by the same random graph model. First, the spectral distribution of each graph is estimated and then, the average spectral distribution of each population is estimated (ρ_*gi*_ (*i* = 1, …, *k*)). Second, the average spectral distribution of all the spectral distributions (ρ_*gM*_) is estimated (average of the average distributions). Finally, the sum of the Kullback-Leibler divergence (KLD) between ρ_*gi*_ (*i* = 1, …, *k*) and ρ_*gM*_ is calculated. Under the null hypothesis, i.e., when all *k* = 3 populations of graphs are generated by the same random graph model, we expect a small Δ.

Figure 1 illustrates the idea behind ANOGVA. In summary, the spectral distribution of each population is compared to the reference distribution (the average of the spectral distributions, ρ_*g*_*M*__). If the sum of the distances (KL divergence) is large, it means that at least one of the spectral distributions is different when compared to the reference (ρ_*g*_*M*__). In other words, at least one of the populations of graphs was generated by a different random graph model and/or set of parameters.

### 2.5. Graph models

#### 2.5.1. Erdös-Rényi random graph

Erdös-Rényi random graph (Erdös and Rényi, 1960) is defined as *n* labeled vertices where each pair of vertices (*v*_*i*_, *v*_*j*_) is connected by an edge with a given probability *p*.

#### 2.5.2. Geometric random graph

A geometric random graph (Penrose, 2003) is constructed by randomly placing *n* vertices in a space R^*d*^ according to a specified probability distribution (usually, uniform distribution) and connecting two vertices by an edge if their distance (according to some metric) is smaller than a certain neighborhood radius *r*.

#### 2.5.3. K-regular random graph

A k-regular random graph (Meringer, 1999) is a graph where every vertex has the same degree (number of adjacent vertices). A k-regular random graph with degree *deg* is called a *deg*-regular random graph or regular random graph of degree *deg*.

#### 2.5.4. Watts-Strogatz random graph

Watts-Strogatz random graph (Watts and Strogatz, 1998) presents small-world properties (short average path lengths) and a higher transitivity (clustering coefficient) than Erdös-Rényi random graphs.

The algorithm for constructing a Watts-Strogatz random graph is as follows:

Input: Let *n*, *nei*, and *p*_*w*_ be the number of vertices, mean degree, and the rewiring probability, respectively.

1. construct a ring lattice with *n* vertices, in which every vertex is connected to its first *nei* neighbors ($\frac{nei}{2}$ on either side);
2. choose a vertex and the edge that connects it to its nearest neighbor in a clockwise sense. With probability *p*_*w*_, reconnect this edge to a vertex chosen uniformly at random over the entire ring. This process is repeated by moving clockwise around the ring, considering each vertex in turn until one lap is completed. Next, the edges that connect vertices to their second-nearest neighbors clockwise are considered. As in the previous step, each edge is randomly rewired with probability *p*_*w*_; continue this process, circulating around the ring and proceeding outward to more distant neighbors after each lap, until each edge in the original lattice has been considered once.

**Output**: the Watts-Strogatz random graph.

#### 2.5.5. Barabási-Albert random graph

Barabási-Albert random graph (Barabasi and Albert, 1999) has a power-law degree distribution due to preferential attachment of vertices (the more connected a vertex is, the more likely it is to receive new edges).

Barabasi and Albert (1999) proposed the following construction: start with a small number of (*n*_0_) vertices. At each iteration, add a new vertex with *m*_1_ (*m*_1_ ≤ *n*_0_) edges that connect the new vertex to *m*_1_ different vertices already present in the graph. To select which vertices the new vertex will connect, assume that the probability that a new vertex will be connected to vertex *v*_*i*_ is proportional to the degree of vertex *v*_*i*_ and the scaling exponent *p*_*s*_ ($P\left(v_{i}\right)\sim degree{\left(v_{i}\right)}^{p_{s}}$, where *degree*(*v*_*i*_) is the number of adjacent edges of vertex *v*_*i*_ in the current iteration.

### 2.6. Simulations description

For the simulation studies, we analyzed five random graph models, namely Erdös-Rényi (Erdös and Rényi, 1960), geometric (Penrose, 2003), k-regular (Meringer, 1999), Watts-Strogatz (Watts and Strogatz, 1998), and Barabási-Albert (Barabasi and Albert, 1999). All simulations were carried out in R using the package igraph. The name of the functions and respective parameters analyzed in our study are: function erdos.renyi.game - parameter p; function grg.game - parameter radius; function k.regular.game - parameter k; function watts.strogatz.game - parameter p; and function barabasi.game - parameter power. For the Watts-Strogatz random graph model, we selected the parameter p as the varying parameter in our simulations because it changes the graph structure without altering the number of edges.

#### 2.6.1. Simulation 1

To verify the controls of types I and II errors of ANOGVA in inferring whether populations of graphs are equally generated, we constructed four scenarios.

1. Scenario 1—under the null hypothesis: We constructed three populations of graphs (*g*_1_, *g*_2_, *g*_3_) with *n* = 300. Graphs were generated by an Erdös-Renyi (ER) random graph model (Erdös and Rényi, 1960). The parameters *p*_*j*_'s (the probability of inclusion of an edge) of the ER random graph models were generated by a truncated normal distribution with lower bound, upper bound, mean and variance set as 0, 10, 1, and 1, respectively, for each graph, and then linearly normalized to the interval [0; 1] (i.e., let *p*_*j*_, *j* = 1, …, |*g*_1_|+|*g*_2_|+|*g*_3_|, be the random numbers generated by the truncated normal distribution. Then, we linearly normalize each *p*_*j*_ to the interval [0; 1] by dividing it by the upper bound). Notice that this normalization is necessary because the parameter of the ER random graph model is the probability of inclusion of an edge. The parameters were generated in the same manner for all graphs of the three populations. This scenario was constructed to evaluate the control of the rate of false positives under the null hypothesis (all graphs were generated by the same random process).
2. Scenario 2—under the alternative hypothesis: Populations of graphs *g*_1_ and *g*_3_ were constructed as described in scenario 1. The parameters of the graphs of population *g*_2_ were generated by a truncated normal distribution with mean 1.5 and unit variance, and then linearly normalized to the interval [0; 1]. This scenario was constructed to evaluate the power of the test when the parameters of the graphs are different.
3. Scenario 3—under the alternative hypothesis: Populations of graphs *g*_1_ and *g*_3_ were constructed as described in scenario 1. The parameters of the graphs of population *g*_2_ were generated by a truncated normal distribution with mean and variance set as 1.5, and 1, respectively, and then linearly normalized to the interval [0; 1]. The number of graphs are set as |*g*_1_| = |*g*_3_| = 125 and |*g*_2_| = 50, 75, 100. This scenario was constructed to evaluate the power of the test when the datasets are not balanced.
4. Scenario 4—under the alternative hypothesis: Populations of graphs *g*_1_ and *g*_3_ were constructed as described in scenario 1. The population *g*_2_ is generated by a Watts-Strogatz random graph model (Watts and Strogatz, 1998) with the dimension of the starting lattice equal to one and the neighborhood within which the vertices of the lattice is connected equal to four. The rewiring probability is generated by a truncated normal distribution with lower bound, upper bound, mean and variance set as 0, 10, 1, and 1, respectively, and then linearly normalized to the interval [0; 1]. The purpose of this scenario is to evaluate the power of the test when one of the populations of graphs is generated by a different random graph model. To make this scenario more realistic, we misclassified the labels of the graphs in rates of 0, 20, 30, and 40%.

For scenarios 1, 2, and 4, the number of graphs varied (|*g*_1_| = |*g*_2_| = |*g*_3_| = 50, 75, 100, 125). For each number of graphs, the experiment was repeated 1,000 times. Then, we constructed receiver operating characteristic (ROC) curves to evaluate the control of the rate of false positives and the power of the proposed statistical test.

#### 2.6.2. Simulation 2

One alternative to verify whether graphs are generated by the same probabilistic process is the application of ANOVA on the features of the graphs (e.g., the betweenness centrality). To compare the performance of ANOGVA with a simple application of ANOVA on the features, we constructed three populations of graphs (*g*_1_, *g*_2_, *g*_3_), each one composed of |*g*_1_| = |*g*_2_| = |*g*_3_| = 100 graphs. The number of vertices was set to *n* = 300. The graphs were generated by Erdös-Renyi (Erdös and Rényi, 1960), geometric (Penrose, 2003), k-regular (Meringer, 1999), Watts-Strogatz (Watts and Strogatz, 1998), and Barabási-Albert (Barabasi and Albert, 1999) random graph models. The parameter of the random graph models were generated by normal distributions with mean one for *g*_1_ and *g*_3_ and mean 1.5 for *g*_2_, all of them with unit variance. Then, they were linearly normalized to the interval [0; 1]. The parameters for these five graph models are: the probability of adding an edge for Erdös-Rényi, the radius for the geometric, i.e., a vertex is connected to all vertices at distance smaller than the radius (we set the space dimension where the vertices are located to two), the vertex degree for the k-regular, the rewiring probability for Watts-Strogatz, and the power of the preferential attachment probability for the Barabási-Albert. Since the parameters for k-regular and Barabási-Albert random graph models are integers, we took the floor of the parameter normalized between zero and one and multiplied it by 10. Then, we measured five features that are commonly considered in the literature, namely the number of edges, the average betweenness centrality (Freeman, 1977) (number of shortest paths from all vertices to all others that pass through that vertex), the average closeness centrality (Bavelas, 1950) (one divided by the sum of the distances from one vertex to all other vertices), assortativity (Newman, 2002) (preference of a vertex to attach to others in terms of degree), and transitivity (Wasserman and Faust, 1994) (relative number of triangles in the graph, compared to total number of connected triples of vertices) for each graph. Finally, we applied ANOVA on these features and compared the statistical power with ANOGVA. A *p*-value cut-off of 0.05 was set to determine whether the test rejected the hypothesis that the three populations were generated by the same random graph model. This experiment was repeated 1,000 times and the proportion of rejected null hypothesis was calculated.

### 2.7. Application to the ABIDE dataset

#### 2.7.1. Dataset description

A large resting state fMRI dataset initially composed of 908 individuals comprising controls and subjects diagnosed with autism and Asperger was downloaded from the ABIDE I Consortium website (http://fcon_1000.projects.nitrc.org/indi/abide/). The ABIDE I dataset is fully anonymized in compliance with the Health Insurance Portability and Accountability (HIPAA) Privacy Rules and the 1,000 Functional Connectomes Project/INDI protocols. Protected health information identifiers and face information from structural images are not included in this dataset. For further details, refer to Di Martino et al. (2014).

To pre-process the brain imaging data, we carried out the Athena pipeline downloaded from (http://www.nitrc.org/plugins/mwiki/index.php/neurobureau:AthenaPipeline) which can be summarized as follows: exclusion of the first four scans; slice timing correction; deoblique dataset; correction for head movements; masking the volumes to exclude non-brain regions; co-registration of mean image to the respective anatomic image of the subject; spatial normalization to MNI space (4 × 4 × 4 mm resolution); extraction of BOLD time series from white matter and cerebrospinal-fluid; removing effects of white matter, cerebrospinal-fluid, motion and trend using linear multiple regression; temporal band-pass filter (0.009 < *f* < 0.08 Hz); and spatial smoothing the filtered data using a Gaussian filter (FWHM = 6 mm). We used the CC400 atlas (Craddock et al., 2012) to define the 351 regions of interest (ROIs). Then, we removed 35 ROIs including the ventricles (identified by using the MNI atlas), resulting 316 ROIs (vertices of the graph) for the construction of functional brain networks. The average time series within the ROIs were considered as to be the region representatives. The head movement during magnetic resonance scanning was treated by using the “scrubbing” procedure described by Power et al. (2012). Individuals with a number of adequate scans less than 100 after the “scrubbing” procedure were discarded. It resulted in 896 subjects for subsequent analyses. Thus, the dataset used in this study is composed of 529 controls (430 males, mean age ± standard deviation, 17.47 ± 7.81 years), 285 autistic patients (255 males, 17.53 ± 7.13 years), and 82 Asperger patients (70 males, 19.97 ± 11.37 years). For further details, see Table 1.

**Table 1:** **Description of the ABIDE data set**.

**Site**	**TR (ms)**	**TE (ms)**	**Voxel-size (mm)**	**Scanner**
Caltech	2,000	30	3.50×3.50×3.50	SIEMENS MAGNETOM TrioTim syngo MR B17
CMU	2,000	30	3.00×3.00×3.00	SIEMENS MAGNETOM Verio syngo MR B17
KKI	2,500	30	3.05×3.15×3.00	PHILIPS Achieva 3T
Leuven	1,667	33	3.59×3.59×4.00	PHILIPS INTERA 3T
MaxMun	3,000	30	3.00×3.00×4.00	SIEMENS MAGNETOM Verio syngo MR B17
NYU	2,000	15	3.00×3.00×4.00	SIEMENS MAGNETOM Allegra syngo MR 2004A
Olin	1,500	27	3.40×3.40×4.00	SIEMENS MAGNETOM Allegra syngo MR 2004A
Pitt	1,500	25	3.10×3.10×4.00	SIEMENS MAGNETOM Allegra syngo MR A30
SBL	2,200	30	2.75×2.75×2.72	PHILIPS INTERA 3T
SDSU	2,000	30	3.43×3.43×3.40	GE 3T MR750
Stanford	2,000	30	3.12×3.12×4.50	GE SIGNA 3T
Trinity	2,000	28	3.00×3.00×3.50	PHILIPS INTERA 3T (conferir)
UCLA	3,000	28	3.00×3.00×4.00	SIEMENS MAGNETOM TrioTim syngo MR B15
UM	2,000	30	3.44×3.44×3.00	GE SIGNA 3T
USM	2,000	28	3.40×3.40×3.00	SIEMENS MAGNETOM TrioTim syngo MR B17
Yale	2,000	25	3.40×3.40×4.00	SIEMENS MAGNETOM TrioTim syngo MR B17

#### 2.7.2. Functional brain networks

A functional brain network can be modeled as a graph, i.e., a pair of sets *G* = (*V, E*), in which *V* is the set of regions of interest—ROIs (vertices), and *E* is the set of functional connectivity (edges) among ROIs. In the current study, the functional connectivity between two ROIs was obtained by calculating the Spearman's correlation coefficient between ROIs *i* and *j* (*i, j* = 1, …, 316) for each individual *q* = 1, …, 896. Thus, a functional brain network *G*^*q*^ with 316 ROIs can be represented by its adjacency matrix **A**^*q*^ with 316 × 316 elements ${\text{A}}_{ij}^{q}$ containing the association between the ROIs *i* and *j* (*i, j* = 1, …, 316; *q* = 1, …, 896). Site, gender, age effects and the proportion of removed volumes by the “scrubbing” were modeled with a generalized linear model (GLM) with the strength of association (*z*-value associated with the Spearman correlation coefficient) as the response variable and the effects as covariates. The residuals of the model were considered as the connectivity filtered by these effects. Then, *p*-values for each Spearman's correlation coefficient (without site, gender, age effects) between ROIs *i* and *j* were calculated and corrected for the false discovery rate (FDR) (Benjamini and Hochberg, 1995). The choice for the Spearman's correlation is based on the fact that it is robust to outliers and is also able to identify non-linear monotonic relationships (de Siqueira Santos et al., 2014).

Functional sub-networks were defined as the same as defined by Sato et al. (2015), namely somatomotor, visual, default-mode, cerebellar, and fronto-parietal.

## 3. Results

### 3.1. Simulations

We evaluated the controls of types I and II errors of ANOGVA in verifying whether graphs are generated by the same random graph model.

**Figure 2:**
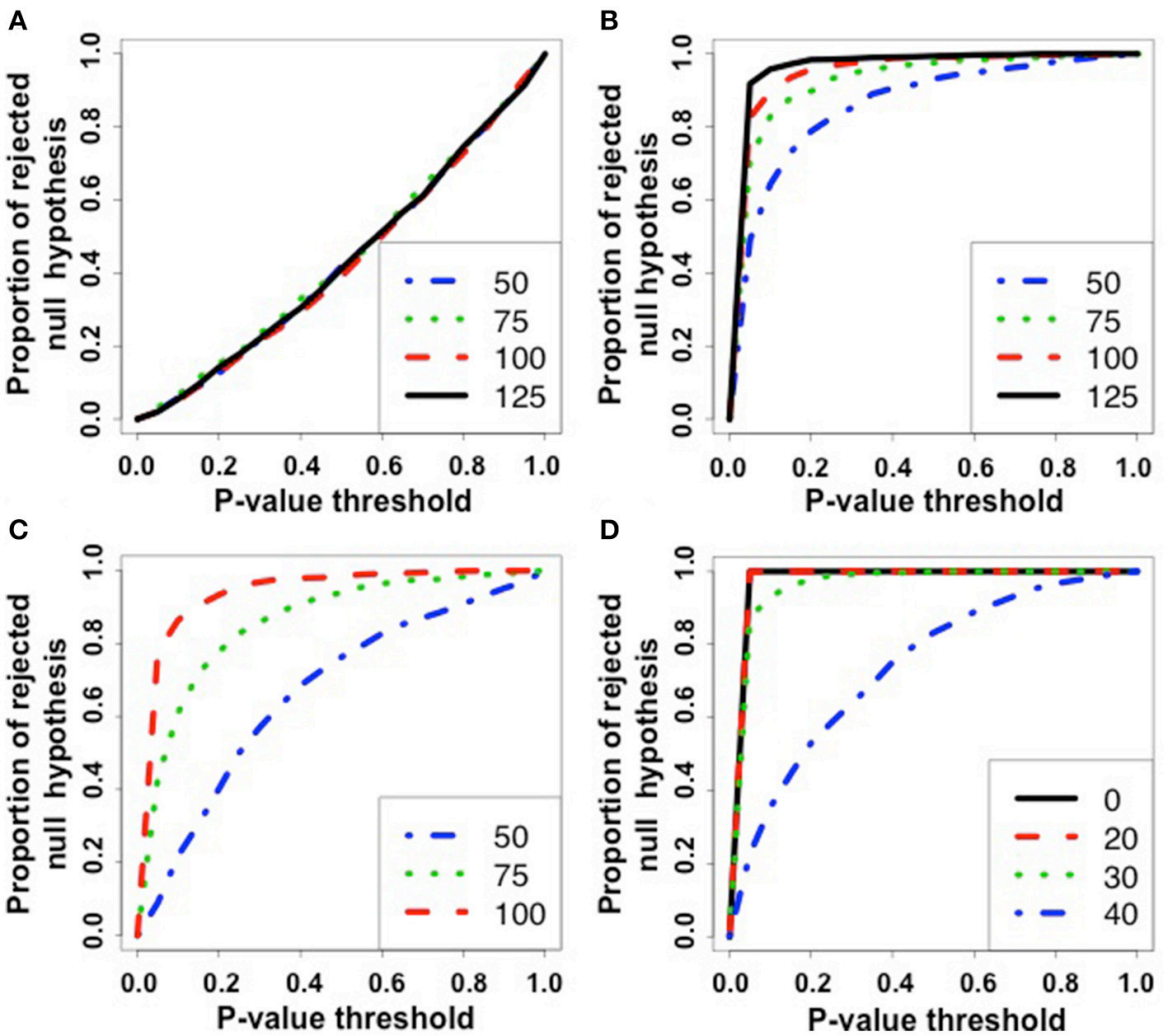
**ROC curves for simulation 1**. The x-axis represents the *p*-value threshold. The y-axis represents the proportion of rejected null hypothesis in 1,000 repetitions. **(A)** Scenario 1: under the null hypothesis. Notice that, under the null hypothesis, the rate of false positives is as expected by the *p*-value threshold. **(B)** Scenario 2: parameters of the graphs are different among populations. Under the alternative hypothesis, the greater the number of graphs (|*g*_1_| = |*g*_2_| = |*g*_3_| = 50, 75, 100, 125), the greater is the power to reject the null hypothesis. **(C)** Scenario 3: unbalanced data. The numbers of graphs are set as |*g*_1_| = |*g*_3_| = 125 and |*g*_2_| = 50, 75, 100. Notice that the more balanced is the number of graphs among populations, the greater is the power. **(D)** Scenario 4: the graph models are different among populations (*g*_1_ and *g*_2_ are Erdös-Rényi and Watts-Strogatz random graph models, respectively) and the labels were misclassified in proportions of 0, 20, 30, and 40%. The greater is the number of mislabeled samples, the lower is the power of the test. Notice that the power when the rate of mislabeling is zero is greater than when the random graph models are equal but the parameter is different.

Figures 2A–D illustrate, respectively, the ROC curves obtained by simulating scenarios 1, 2, 3, and 4 described in Section 2.6.1. The x-axis represents the *p*-value threshold and the y-axis represents the proportion of rejected null hypothesis given a *p*-value threshold. The ROC curve under the null hypothesis lies in the diagonal. Under the alternative hypothesis, we expect to obtain a curve above the diagonal. In our case, the nominal *p*-value is on the x-axis and the proportion of rejected null hypotheses is on the y-axis.

The further is the curve above the diagonal, the higher is the power of the test. By analyzing the ROC curves, it is possible to notice that (i) Figure 2A—the ROC curves under the null hypothesis lie in the diagonal as expected, i.e., the statistical test is effectively controlling the rate of false positives (the proportion of rejected null hypothesis is as expected by the *p*-value threshold); (ii) Figure 2B—the power of the test increases as the number of graphs increases under the alternative hypothesis (the parameter of one of the populations is generated in a different manner); (iii) Figure 2C—the power of the test increases as the datasets are more balanced; and (iv) Figure 2D—the case the graph samples are generated by different models, the power of the test increases as the number of misclassified graphs decreases. When the number of misclassification is zero, it is possible to notice that the power of the method is higher than when only the parameter is different (Figure 2C).

**Figure 3:**
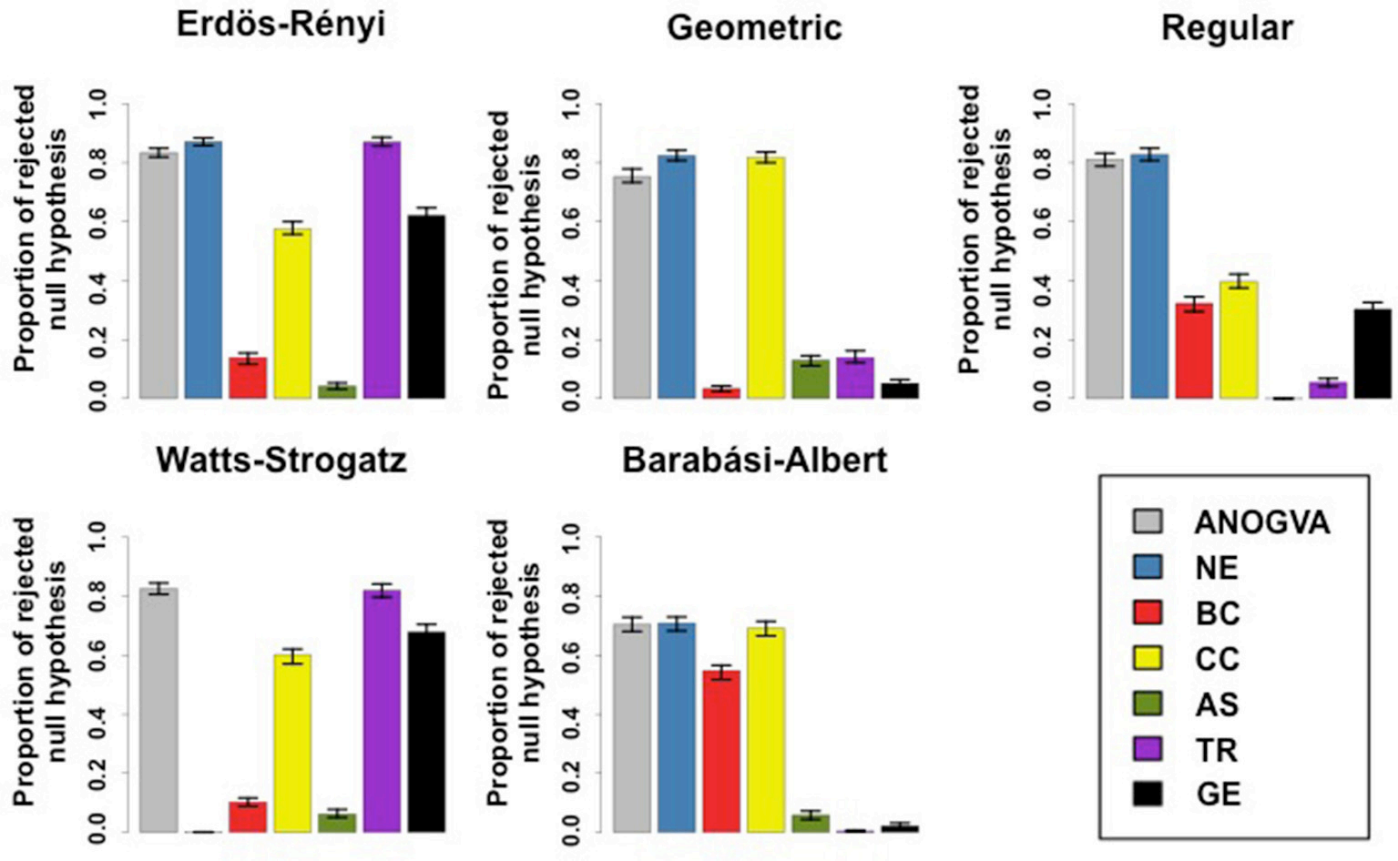
**Proportion of rejected null hypotheses at a *p*-value threshold of 0.05 in 1,000 repetitions**. Error bars indicate the 95% confidence interval. Each color represents the ANOGVA (gray) or the analyzed feature with ANOVA (NE, number of edges; BC, Betweenness centrality; CC, closeness centrality; AS, assortativity; TR, transitivity or clustering coefficient; GE, global efficiency).

We compared ANOGVA with the application of ANOVA on other features, such as the number of edges, betweenness centrality, closeness centrality, assortativity, and transitivity in five random graph models namely, Erdös-Rényi, geometric, k-regular, Watts-Strogatz, and Barabási-Albert. Figure 3 describes the proportion of rejected null hypotheses in these graph models. By analyzing Figure 3, we notice that only ANOGVA followed by transitivity are able to discriminate all random graph models with different parameters, including the Watts-Strogatz random graph model. Betweenness centrality presented low power for geometric, k-regular, Watts-Strogatz, and Barabási-Albert random graph models. Closeness centrality was not able to identify differences between Watts-Strogatz random graph models. Assortativity coefficient presented low power for Erdös-Rényi and Watts-Strogatz random graph models.

### 3.2. Autism spectrum disorder

**Figure 4:**
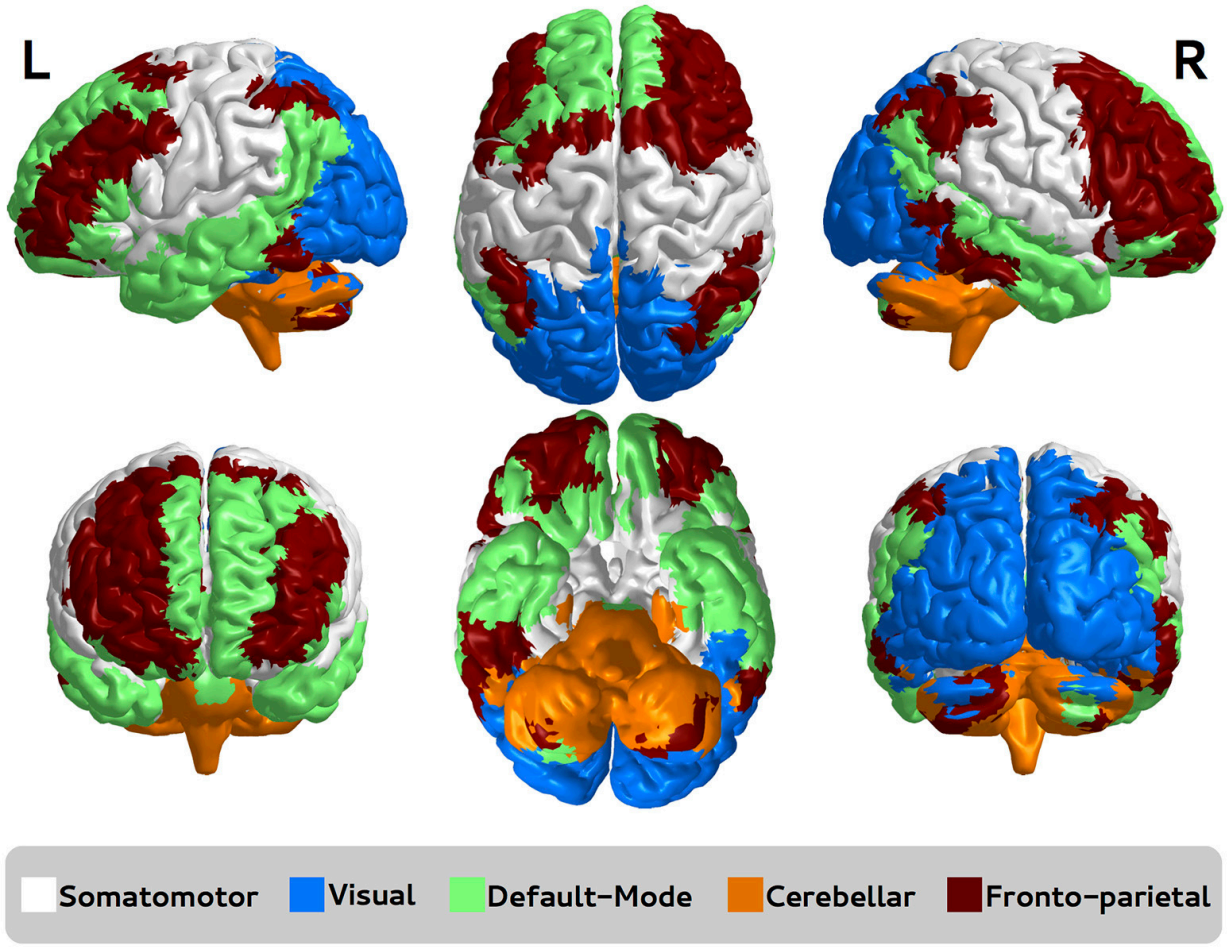
**Functional brain sub-networks defined by Sato et al. (2015)**. Each sub-network is represented by a different color, namely somatomotor, visual, default-mode, cerebellar, and fronto-parietal. R, right; L, left.

Functional brain networks were constructed as described in Section 2.7.2. The five sub-networks depicted in Figure 4 are based on sub-networks defined by Sato et al. (2015), namely somatomotor, visual, default-mode, cerebellar, and fronto-parietal.

Here, we focused on the identification of which sub-network is associated with autism and Asperger by using ANOGVA. The number of permutations was set to 1,000. First, we compared the three groups (controls vs. autism vs. Asperger) for each sub-network to verify if there is at least one population that differs from the others. The test indicated a significant difference at a *p*-value threshold of 0.05 for cerebellar (*p* = 0.04), and not for somatomotor (*p* = 0.13), visual (*p* = 0.95), default-mode (*p* = 0.17), and fronto-parietal (*p* = 0.21). These results suggest that the structure of the cerebellar functional sub-network is different at least in one of the populations among controls, autistic and Asperger patients. To identify which population is not equally generated in the cerebellar cluster, we carried out pairwise comparisons among the groups. Results indicate that there is no statistical evidence to discriminate controls vs. Asperger (*p* = 0.99) and autism vs. Asperger (*p* = 0.13), but there is significant difference between controls and autism (*p* < 0.001). This result indicates that the random process underlying the group control and autism are different.

## Discussions

We introduced a method that is able to test two or more groups of graphs simultaneously. Our permutation-based test allows the estimation of *p*-values even for datasets with unknown probability distributions.

In a simulation study, we showed that the proposed method can indeed discriminate samples of graphs generated by different models and parameters. Similar to ANOGVA, the application of ANOVA on the number of edges can also discriminate a wide range of graph models. However, when the number of edges do not change, such as in the case of the Watts-Strogatz random graph model (the parameter is the rewiring probability), the ANOVA on the number of edges fails. Notice that the parameter of the Watts-Strogatz model only changes the structure of the graph and, in general, graph features are associated with the number of edges.

In the illustrative fMRI application example, we found that the cerebellar is associated with autism spectrum disorder. This result is consistent with other findings reported in the literature (Fatemi et al., 2012; Becker and Stoodley, 2013). Sato et al. (2015) showed that the network entropy of the cerebellar system is lower in autism than controls. Other fMRI studies reported reduced connectivity within cerebro-cerebellar motor networks during finger sequence tapping (Mostofsky et al., 2009) and also related to verb generation (Verly et al., 2014). Mosconi et al. (2015) showed that feedforward and feedback motor control abnormalities implicate cerebellar dysfunctions. We didn't find statistical differences between control and Asperger groups and between autism and Asperger groups. These results do not mean that there are no differences in graph structures between these groups as there is evidence in the literature that the brain activity and structure between these groups can be distinguished (McAlonan et al., 2002; Welchew et al., 2005). This result is most probably a result in lack of statistical power that can be improved by increasing the sample size, improving the classification criteria of the groups, and/or changing the experimental setup.

One limitation of our study is that the data collection protocols are heterogeneous among labs belonging to the ABIDE consortium. This issue was addressed by including the site as covariate in the GLM. Another solution would be to select the lab presenting the greater number of samples. However, since the number of samples decrease considerably when compared to analyzing the entire ABIDE dataset, the power of the test also decreases considerably. Another limitation is the fact that the results are based on a resting-state fMRI protocol, and caution has to be taken to extend to other cognitive states. Regarding the method, ANOGVA can only be applied to undirected graphs. For directed graphs, more studies of the spectrum are necessary. Since the statistical test is based on a permutation procedure, one supposition is that the parameters of the model are sampled from probability distributions with finite variance.

ANOGVA is implemented in R (R Core Team, 2014) and is available in the package statGraph (http://www.ime.usp.br/~fujita/software.html).

## Author contributions

AF and DT conceived the experiments, analyzed the results, and drafted the manuscript. AF conducted the experiments. MV pre-processed the fMRI data. All authors gave the final approval for publication.

## Funding

AF was partially supported by São Paulo Research Foundation (FAPESP 2013/01715-3, 2013/03447-6, 2013/07375-0, 2015/01587-0, and 2016-13422-9), CNPq (306319/2010-1), and NAP eSciencePRPUSP. MV was partially supported by CAPES fellowship. DT was partially supported by Pew Latin American Fellowship and Ciência Sem Fronteiras Fellowship (CNPq 246778/2012-1).

### Conflict of interest statement

The authors declare that the research was conducted in the absence of any commercial or financial relationships that could be construed as a potential conflict of interest.
